# NbO_x_ Mott Memristor‐Based Oscillatory P‐trit for Ternary Potts Machine

**DOI:** 10.1002/advs.76754

**Published:** 2026-07-27

**Authors:** Hakseung Rhee, Seoeun Jang, Tae Wook Go, Woojoon Park, Gwangmin Kim, Do Hoon Kim, Hanchan Song, Younghyun Lee, Junmo Kang, Daehee Kim, Kyung Min Kim

**Affiliations:** ^1^ Department of Materials Science and Engineering Korea Advanced Institute of Science and Technology (KAIST) Daejeon Republic of Korea; ^2^ Graduate School of Semiconductor Technology Korea Advanced Institute of Science and Technology (KAIST) Daejeon Republic of Korea; ^3^ Peter‐Grünberg‐Institut 14 (PGI‐14) Forschungszentrum Jülich GmbH Jülich Germany; ^4^ Peter‐Grünberg‐Institut 7 (PGI‐7) Forschungszentrum Jülich GmbH Jülich Germany; ^5^ Electronics and Telecommunications Research Institute (ETRI) Daejeon South Korea; ^6^ Center for Semiconductor Technology Korea Institute of Science and Technology (KIST) Seoul Republic of Korea

**Keywords:** combinatorial optimization, energy‐based model, niobium oxide, potts machine, probabilistic computing, p‐trit, threshold switching memristor

## Abstract

Probabilistic computing is an emerging approach for solving combinatorial optimization and probabilistic inference by leveraging stochastic dynamics to explore complex energy landscapes. However, most hardware implementations remain constrained to binary probabilistic bits (p‐bits), limiting efficiency for inherently multi‐class problems. Here, we propose and experimentally demonstrate a ternary Potts machine (TPM) enabled by a NbO_x_ oscillator‐based probabilistic ternary digit unit (i.e., p‐trits). The p‐trit exploits electro‐thermal dynamics of a NbO_x_ threshold‐switching memristor that exhibits two distinct negative differential resistance regimes, producing stochastic transitions among three discrete states (off/osc/on). A compact p‐trit circuit digitizes the oscillatory signals into two‐bit outputs, yielding a practical p‐trit with an operation rate of 2.5 µs/bit, while the NbO_x_ oscillator, which is the primary source of energy consumption, consumes 2.4 nJ/bit. A microcontroller unit ‐integrated TPM prototype solves Max‐3‐Cut with ∼30% higher accuracy than p‐bit‐based computing while requiring ∼33% of its computational overhead. Simulations on the weighted ternary number partitioning problem confirm scalability and robustness, showing convergence of up to 99.96%, comparable to near‐optimal solutions.

## Graphical abstract

A ternary Potts machine utilizing NbO_x_‐based probabilistic trits (p‐trits) is demonstrated for efficient combinatorial optimization. By leveraging the electro‐thermal switching dynamics of NbO_x_ memristors, this architecture generates three‐state stochastic fluctuations. This approach significantly outperforms binary counterparts in accuracy and computational efficiency, marking a key advancement toward scalable multinary probabilistic computing.

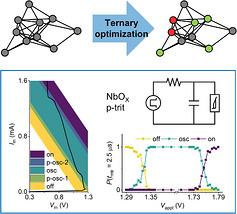

## 1 Introduction

Energy‐based models (EBMs) provide a rigorous computational framework for solving complex optimization problems and learning high‐dimensional probability distributions. EBMs assign scalar energy to each system state and seek directions to minimize this energy, thereby navigating rugged energy landscapes. The Boltzmann machine (BM) is a representative implementation of EBMs, which employs stochastic sampling to search for the minimum‐energy configuration, i.e., the most probable solution [1]. Recently, researchers have actively pursued hardware implementations of BMs to provide compact, energy‐efficient, and CMOS‐compatible solutions that can be seamlessly integrated into existing semiconductor systems for IoT and edge devices. Such BM hardware has primarily utilized probabilistic bits (p‐bits), which are elementary devices capable of defining the appearance probability of binary digits (0 or 1). Therefore, various types of p‐bits have been developed, including magnetic tunnel junction‐based [2, 3, 4], memristor‐based [5, 6, 7], and CMOS‐based circuits [8] and devices [9, 10]. Although their operating mechanisms differ, they all commonly exploit the probabilistic behavior of energetically similar bistable systems. Since bistable systems are common in physical domains, continued development of diverse p‐bit devices is expected.

However, computing systems can solve more complex problems more efficiently when computation is performed using multinary computational digits [11, 12, 13, 14, 15]. In the case of BM, when each of *N* nodes can occupy *k* states instead of 2, the size of the solution space grows exponentially from 2^*N*^ to *k^N^*, thereby enabling the modeling of a richer energy landscape. Such multinary extensions are theoretically supported by the Potts model, a generalization of the Ising model in statistical physics [16, 17, 18, 19, 20, 21]. In this formulation, each node is represented as a unit vector in a two‐dimensional plane, parameterized by an angle θ∈{0,2π/k,4π/k,…}. Then, the Hamiltonian *H*(*x*)is expressed as;

(1) $$H\left(x\right)=-\sum_{i<j}J_{ij}\;x_{i}\cdot x_{j}-\sum_{i}h_{i}x_{i}$$

where ***x**_i_* denotes the spin vector of node *i* defined as ***x**_i_* =  (cos θ_*i*_, sin θ_*i*_), *J_ij_* represents the coupling coefficient between nodes *i* and *j*, and ***h**_i_* is a bias vector of ***x**_i_*. The hardware implementation of the Potts model necessitates physical systems that can realize multinary digits, yet no hardware leveraging this principle has been demonstrated to date.

The electro‐thermal dynamics in a NbO_x_‐based oscillator are one of the well‐known high‐order dynamic systems [22, 23, 24, 25, 26]. The oscillator incorporates NbO_x_‐based threshold switching memristors (TSM) that exhibit two distinct negative differential resistance (NDR) behaviors; the NDR at lower current levels is induced by field‐triggered thermal runaway at low current, which arises from Joule‐heating‐assisted conductivity enhancement under an applied electric field [27, 28]. The second NDR at higher current levels originates from a Mott‐transition‐related insulator–metal transition, leading to an abrupt change from a high‐resistance insulating state to a low‐resistance metallic state [27, 29, 30]. These NDR behaviors arise from bistability activated under specific bias conditions, where transitions between the two states occur stochastically due to thermal noise. The oscillator configuration with the NDR device (referred to as the NbO_x_ oscillator) provides an electrical means to precisely monitor these transition processes. When configured as an oscillator, three deterministic states can be obtained based on the two NDR boundaries: a non‐oscillating off state, an oscillatory state where on and off transitions alternate, and a non‐oscillating on state. The boundaries between these deterministic states can operate in a probabilistic manner, suggesting that the oscillator can serve as a physical platform for implementing the Potts model.

In this study, we propose and experimentally demonstrate a ternary Potts machine (TPM) that leverages the NbO_x_ oscillator‐based p‐trit as its fundamental computational unit. This work includes the approximation of the probabilistic sampling rule, the design of a p‐trit for efficient probability extraction, and the fabrication and evaluation of a TPM hardware prototype (a schematic overview of the overall system flow is provided in Figure S1).

First, we introduce the segmented probabilistic sampling (SPS) rule, an approximate probabilistic sampling method required for implementing a probabilistic TPM in hardware. This rule provides a theoretical basis for extending conventional Boltzmann machines to TPMs and is particularly compatible with hardware implementation. Next, we show that the probability values required to implement the SPS rule can be effectively obtained using the first‐time‐to‐oscillate (FTTO) method. As a result, the proposed p‐trit reliably distinguishes three operational regimes with two stochastic boundaries, enabling a compact and physically implementable ternary probabilistic unit.

Next, we validate the system‐level feasibility of the proposed framework using a lab‐scale TPM hardware prototype integrated with a microcontroller unit (MCU)‐based virtual p‐trits for solving the Max‐3‐Cut (M3C) problem. The results confirm that the TPM outperforms the conventional BM, achieving ∼30% higher accuracy while requiring only ∼33% of the computational cost. Subsequently, the scalability and robustness of the proposed TPM are evaluated through simulations of the high‐dimensional weighted ternary number partitioning problem, suggesting its feasibility as a core unit of an energy‐efficient and scalable hardware solver for large‐scale optimization problems.

## 2 Results

### 2.1 Theoretical Framework of Probabilistic Potts Model With Segmented Probabilistic Sampling (SPS) Rule

**FIGURE 1:**
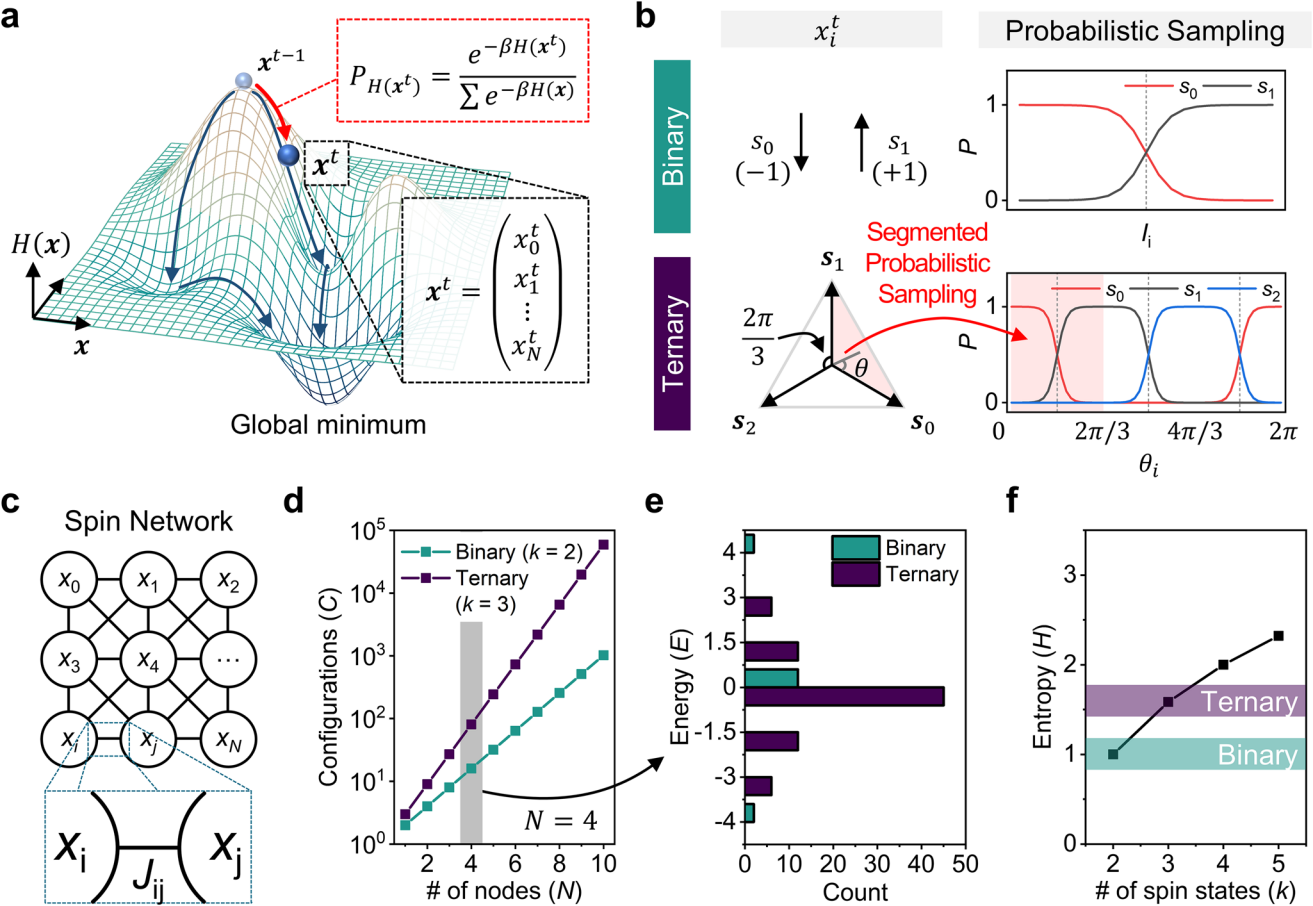
Comparison of ternary Potts machine and binary Boltzmann machine. (a) Conceptual illustration of Boltzmann sampling in an arbitrary energy landscape. Probabilistic update rules ($P_{H(x^{t})}$) guide the solution vector ***x** ^t^* toward the global minimum. (b) Vector representation of states and probabilistic update rules for the binary p‐bit (upper) and ternary p‐trit (lower). (c) Schematic of a spin network with a size of *N*, where each node interacts via a coupling matrix ***J***. (d) Total number of configurations (*C*) representable for network size *N*. (e) Distribution of the discrete energy states (*E*) for an arbitrary network with a size of *N* = 4. (f) Shannon entropy (*H*) per node as a function of the number of node states (*L*).

Figure 1a illustrates an example of an energy landscape of the Hamiltonian *H*(***x***) as a function of the high‐dimensional state vector $x^{t}=\left(x_{0}^{t},x_{1}^{t},\text{…},x_{N}^{t}\right)$ at time *t*. For intuitive understanding, the high‐dimensional configuration space is projected onto a two‐dimensional plane, where each point ***x** ^t^* represents one configuration, and the vertical axis denotes the corresponding energy *H*(***x** ^t^*). At a given time *t*, the state transition from ***x*** ^*t* − 1^ to ***x** ^t^* occurs according to the following Boltzmann distribution, where lower energy states have higher selection probabilities;

(2) $$P_{H\left(x^{t}\right)}=\frac{e^{-\beta H\left(x^{t}\right)}}{\sum e^{-\beta H\left(x\right)}}\;$$

where β  =  1/*kT*, *k* is a Boltzmann constant, and *T* is the temperature variable. The model explores the state space through stochastic sampling, progressively moving the state toward the lower energy configurations.

Here, as the available number of states for the node *x_i_* increases, the representational capacity of the energy landscape is enhanced. Figure 1b compares two models: a conventional Boltzmann machine (BM, upper panel) and a ternary Potts machine (TPM, lower panel). The BM consists of probabilistic binary nodes known as p‐bits. Each p‐bit node *x_i_* can take on either +1 or −1 as its value, which is determined probabilistically based on an input *I_i_*. The probability distribution of *x_i_* being +1 follows a sigmoid function defined as:

(3) $$P_{\;x_{i}=+1}=\frac{1}{1+e^{-\beta I_{i}}}\;$$

where $I_{i}=-\frac{\partial H}{\partial x_{i}}$. Note that $P_{\;x_{i}=+1}=1-P_{\;x_{i}=-1}$. This probabilistic sampling rule ensures that the system evolves over time toward a state distribution that converges to the Boltzmann distribution (Note S1).

Compared to the BM, the TPM uses the p‐trits. Based on the vector Potts model, the three states can be represented as three unit vectors uniformly separated by 2π/3 defined as *x_i_* =  (cos θ_*i*_, sin θ_*i*_) in a two‐dimensional plane, where $\theta_{i}\in \left\{0,\;\frac{2\pi }{3},\;\frac{4\pi }{3}\right\}$. Unlike scalar representations (e.g., {0, 1, 2} or {–1, 0, +1}), this vector representation ensures that all pairwise spin interactions are energetically equivalent, enabling the construction of the ternary Potts Hamiltonian through vector inner products.

While the Potts model defines the energy of multi‐state interactions, it does not include a sampling rule that describes how the states are probabilistically updated. To address this, we newly propose the *Segmented Probabilistic Sampling* (SPS) rule, in which probabilistic sampling occurs only between two neighboring states based on the input level (Note S2). The rationale for this approximate sampling rule can be briefly explained as follows. The three discrete states of a ternary Potts spin are represented by equally spaced two‐dimensional unit vectors, allowing the entire ternary state space to be parameterized by a single input variable θ_*i*_. Assuming that the system follows a Boltzmann distribution, the transition probability can be approximated by a sigmoidal probabilistic transition between the two neighboring states closest to θ_*i*_, whereas the transition to the farthest state is negligible because of its prohibitively high energy cost. In this way, the SPS rule defines probabilistic transitions only between adjacent states and effectively extends probabilistic sampling in BM to a three‐state framework by confining the probabilistic update to local angular intervals (Note S3).

The probability of *x_i_* at the phase state $\theta_{i}=\frac{2n\pi }{3}$​ is given by:

(4) $$P_{\;\theta_{i}=2n\pi /3}=\frac{1}{1+e^{-\beta^{\prime }I_{i}}}\;,\;I_{i}\in \left[\frac{2\left(n-1\right)\pi }{3},\;\frac{2n\pi }{3}\right),\;n\in \left\{1,\;2,\;3\right\}$$

Here, $I_{i}=-\frac{\partial H}{\partial \theta_{i}}$, and $\beta^{\prime }=\frac{2\pi }{3k_{B}T}$. Note that in each of the three phase intervals, $\left(0,\;\frac{2\pi }{3}\right],\;\left(\frac{2\pi }{3},\;\frac{4\pi }{3}\right],\;\left(\frac{4\pi }{3},\;2\pi \right]$, only two adjacent phase states have non‐zero probability, while the third state is excluded. For example, in the interval $\left(0,\;\frac{2\pi }{3}\right]$, the probability of the phase state $\theta_{i}=\frac{2\pi }{3}$​ is defined as in the above equation, while the other two probabilities are given by $P_{\;\theta_{i}=0}=1-P_{\;\theta_{i}=2\pi /3}$ and $P_{\;\theta_{i}=4\pi /3}=0$. This sampling rule only requires defining the probabilities between two neighboring states, making it applicable to more complex multinary systems if the probabilistic multinary unit is provided.

The BM or TPM system can be illustrated using the simplest form of a spin network, as shown in Figure 1c. In this network, node pairs *x_i_* and *x_j_* are coupled via interaction coefficients *J_ij_*. If each node can represent *k* discrete states, a network of *N* nodes yields a configuration (*C*) of size *k^N^*. Therefore, as shown in Figure 1d, ternary systems achieve an exponentially larger capacity than their binary counterparts. Such a higher capacity enables the system to represent a richer energy landscape for a given problem. Figure 1e shows the energy state distribution of the spin network with *N*  =  4 for all possible configurations, calculated from the energy $E\;\left(x\right)=\sum_{i,j}J_{ij}x_{i}x_{j}$, using the arbitral interaction matrix ***J***,

(5) $$J=\left(\begin{array}{cccc} 0 & -1 & 0 & 1\\ -1 & 0 & 1 & 0\\ 0 & 1 & 0 & -1\\ 1 & 0 & -1 & 0 \end{array}\right)\;$$

The count represents the number of configurations that yield the energy value. The total counts are 2^4^ for the binary system and 3^4^ for the ternary system. In this example, the binary system can represent only three distinct energy levels over an energy range (from +4 to −4), whereas the ternary system yields five energy levels, indicating that the ternary one provides a more richer energy states for the given network.

To further quantify the multi‐state systems, we analyze Shannon entropy ($H=\sum_{i=0}^{L-1}-p_{i}\log_{2}p_{i}$) of those systems, which provides a measure of the uncertainty or information content associated with a probabilistic distribution [31]. In this context, a higher entropy indicates that the system can explore a wider variety of states and thus requires more information to fully describe its configuration. Assuming a uniform distribution over all states, the maximum entropy per node is log _2_ *k*, where *k* is the number of states per node, and the total entropy of the system with *N* nodes becomes *H*  =  *N* × log _2_ *k*. As shown in Figure 1f, the ternary system achieves approximately 1.58 bits per node, compared to 1 bit in the binary system, highlighting its greater expressiveness and higher information density at the same network size.

### 2.2 Efficient Probability Extraction from NbO_x_ Oscillator Using First‐Time‐To‐Oscillate (FTTO) Metric

**FIGURE 2:**
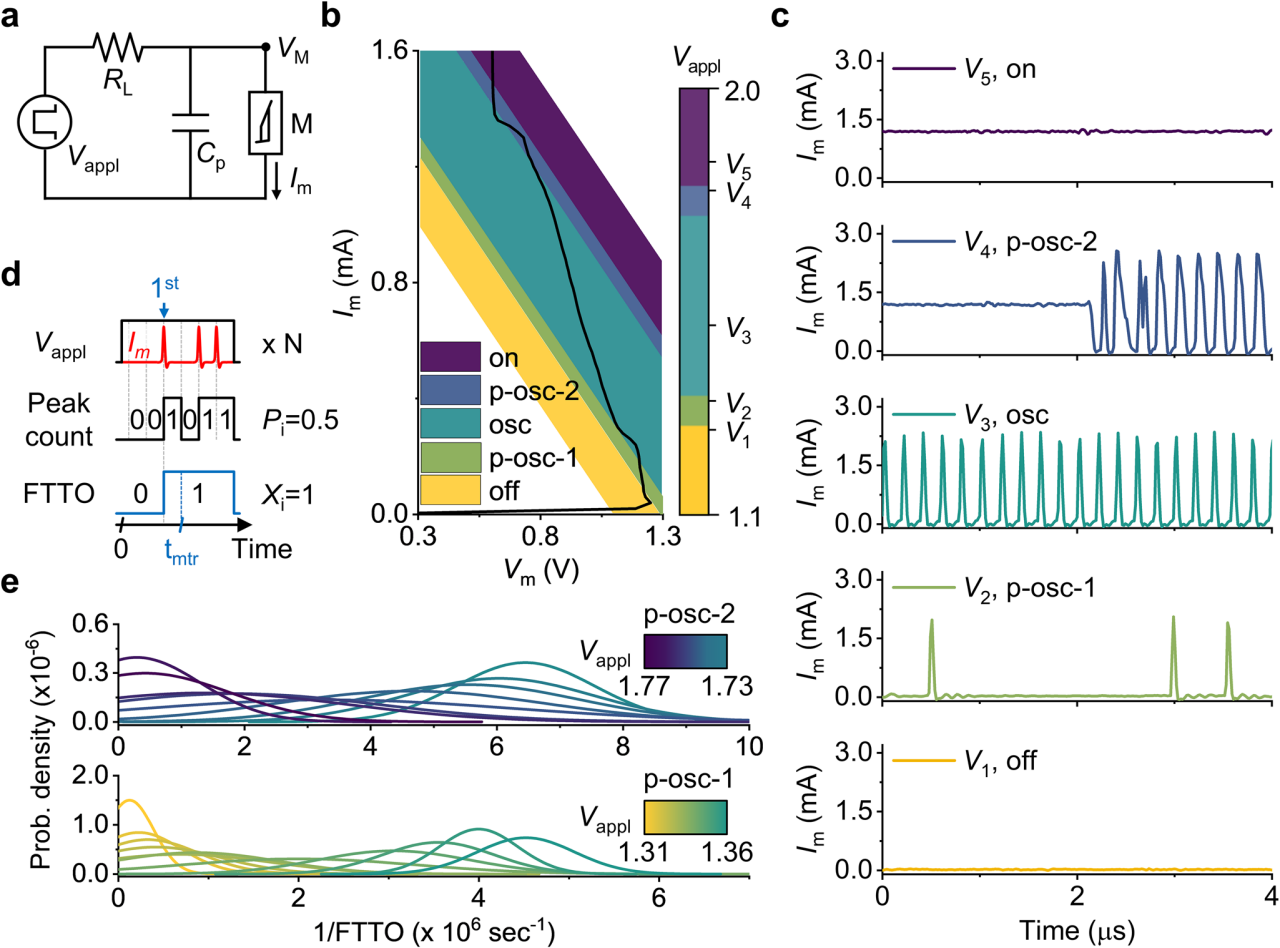
Probabilistic ternary regimes in the NbO_x_ oscillator. (a) A circuit diagram of the NbO_x_ oscillator. (b) *I–V* curve of NbO_x_ TSM with two NDR regions (black line) and corresponding five states (colored regions): three deterministic states, off, osc, and on, and two probabilistic states, p‐osc‐1 and p‐osc‐2. (c) Examples of time‐domain responses for each state. (d) Comparison of sampling methods: the conventional peak number counting‐based method (middle panel), and the proposed FTTO‐based method (bottom panel) for the identical probabilistic signal (top panel). (e) 1/FTTO distributions for p‐osc‐1 (top) and p‐osc‐2 regions (bottom) with various *V_appl_*.

The NbO_x_ oscillator can implement probabilistic ternary regimes according to the SPS rule. As shown in Figure 2a, the NbO_x_ oscillator consists of a NbO_x_ TSM (denoted as M), a series load resistor *R_L_*, and a parallel capacitor *C_P_* (see Figure S2 for the capacitance configuration and values). The TSM with a TiN/NbO_x_/TiN structure exhibited two NDR regions in its current‐controlled current‐voltage (*I–V*) curve (Figure 2b) [32]. When combined with the circuit components (*R_L_*, *C_p_* ​), the NDR device constitutes a Pearson–Anson oscillator, whose operating regime can be controlled by the applied voltage conditions. When *R_L_* of 800 Ω was used (selected based on load‐line analysis to balance the probabilistic operating windows, Figure S3), five distinct circuit‐level operating regimes could be defined depending on the applied voltage: three deterministic regimes; off, periodic oscillation (osc), and on, and two probabilistic regimes; one between off and osc (p‐osc‐1) and the other between osc and on (p‐osc‐2) (the regime boundaries are also influenced by the effective parallel capacitance; see Figure S4). Although the off and on regimes are fixed equilibrium states, whereas the oscillation regime is a limit cycle, all three correspond to stable attractors of the Pearson–Anson oscillator and can therefore be treated as discrete and equivalent system‐level regimes. Figure 2c shows five representative current outputs at the applied voltage (*V_appl_*) of *V*_1_ = 1.290 V, *V*_2_ = 1.340 V, *V*_3_ = 1.500 V, *V*_4_ = 1.785 V, *V*_5_ = 1.850 V. As the *V_appl_* increased, the states changed in the order of off, p‐osc‐1, osc, p‐osc‐2, and on. The origin of these probabilistic states is attributed to electrical and thermal noise perturbations [6, 33, 34, 35, 36, 37, 38, 39].

To utilize this device for p‐trits, the probabilities between neighboring deterministic regimes must be extracted within the probabilistic operational region. Here, we propose a novel approach to quantify the probability via the *First‐Time‐To‐Oscillate* (FTTO) metric, defined as the time interval from the start of *V_appl_* to the first oscillation peak. Figure 2d compares the conventional peak number counting‐based probability quantification method (middle panel) and the proposed FTTO‐based (bottom) for a given oscillation waveform (top). In conventional peak‐counting methods, the oscillatory output must be continuously monitored over a predefined observation window, and the number of oscillation events should be counted to estimate the probability. This process generally requires additional functional blocks such as peak‐detection circuitry, clocked counters, memory/register units for count storage, and digital post‐processing logic [6]. Additionally, this method becomes difficult to accurately obtain the probability when burst pulses occur (as shown in Figure 2c for *V*_4_ = 1.785 V) or when oscillations exhibit high autocorrelation. Moreover, the oscillation speed varies with voltage even within the oscillation region, making it challenging to accurately determine the timing for peak detection (Note S4). In contrast, the proposed FTTO metric only detects whether the first oscillation occurs within a monitoring window (*t_mtr_*), enabling a simpler readout architecture based on threshold detection and latch/D‐FF operation without requiring continuous oscillation counting. Furthermore, this probabilistic behavior can be tuned by adjusting the *t_mtr_*. Specifically, the stochastic transition rate λ(*V_appl_*) at each NDR boundary is an intrinsic device characteristic, while *t_mtr_* determines the cumulative transition probability within the observation window as $P=1-e^{-\lambda \left(V_{appl}\right)\cdot t_{mtr}}$. A larger *t_mtr RS‐2026‐25542514_* broadens the sigmoidal *P_osc_* −*V_appl_* profile, providing a practical degree of freedom to optimize the sampling characteristics for a given computational task.

An increase in oscillation probability corresponds to a shorter FTTO. Therefore, the FTTO metric can provide the *V_appl_*‐dependent probability values. Figure 2e shows the experimentally obtained inverse‐FTTO (1/FTTO) distributions characterized by their mean and standard deviation, for various *V_appl_* for the two probabilistic oscillation regimes; from 1.310 to 1.360 V for p‐osc‐1, and from 1.770 to 1.730 V for p‐osc‐2, each in 5 mV steps. Each distribution was obtained from 50 measurements with 10 µs pulses. If no oscillation occurred during the pulse duration, the FTTO was regarded as infinity (i.e., 1/FTTO = 0). In both *V_appl_* ranges, as the *V_appl_* approaches the oscillation state (the higher *V_appl_* in the p‐osc‐1 state and the lower *V_appl_* in the p‐osc‐2 state), FTTO decreases, producing a distribution with higher 1/FTTO values. By integrating the 1/FTTO probability distribution over *t_mtr_*, the probability that an event has occurred can be obtained, which is discussed in the following section.

### 2.3 P‐trit Circuit Design and Experimental Validation

**FIGURE 3:**
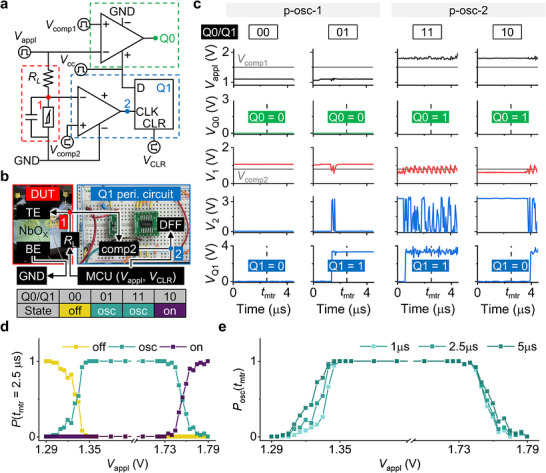
Circuit implementation of NbO_x_ p‐trit. (a, b) Circuit diagram of NbO_x_ p‐trit (a) and experimental setup for Q0 and Q1 (b). The table summarizes the Q0/Q1​ output states corresponding to the ternary regimes. (c) Examples of node voltage responses for four states of Q0/Q1. (d, e) Probability distribution of off, osc, and on states obtained at *t_mtr_* of 2.5 µs (d) and various *t_mtr_* as a function of *V_appl_* (e).

We proposed a NbO_x_ p‐trit unit as shown in Figure 3a, which generates two‐digit outputs from the probabilistic FTTOs of the NbO_x_ oscillator. This circuit consists of three parts: an oscillator (red dashed box), an input‐level segmentation part (green dashed box), and a first‐oscillation detection part (blue box). Figure 3b shows a breadboard‐based prototype for experimental validation. The input voltage was applied through an MCU, and the NbO_x_ device under test (DUT) was loaded on a probe station and connected to the breadboard via cables. During operations, the voltage levels of each colored node were monitored by an oscilloscope. Details about the components used are described in the Methods section. The p‐trit circuit returns two digital output values, Q0 and Q1, to represent three states, where Q0 indicates whether the deterministic state is on or off, and Q1 determines whether oscillation has occurred. For Q0, the node *V_appl_* is connected to the V– terminal of the comparator, with a reference pulse (*V*_*comp*1_, 1.5 V) applied to its V+ terminal. Therefore, the output of this comparator (Q0) determines whether the initial state is on or off by comparing the *V_appl_* and *V*_*comp*1_. For Q1, node 1 is connected to the V– terminal of the other comparator, with a reference pulse (*V*_*comp*2_, 0.8 V) to its V+ terminal. This configuration amplifies the small analog signal of node 1 to the digital output of node 2. This digital output serves as a clock input for a D flip‐flop (DFF), which detects the rising edge of the first oscillation event and remains latched thereafter. The clear pulse (*V_CLR_*) is applied before *V_appl_* by the MCU. Consequently, the DFF output (Q1) determines whether oscillation has occurred. As a result, the combination of Q0 and Q1 defines the p‐trit states: (00) = off, (01) or (11) = osc, and (10) = on, which are assigned to the corresponding Potts spin vectors with angles θ_*i*_ =  0,  2π/3,  4π/3, respectively. It should be noted that, in our p‐trit, the symmetry required by the Potts model does not originate directly from the oscillator regimes themselves. Instead, it is guaranteed by assigning the digital outputs derived from the oscillator regimes to the corresponding Potts spin vectors as labels and subsequently mapping them onto the mathematical structure of the vector representation.

Figure 3c shows the experimental results, showing examples of all four possible Q0/Q1 combinations. Here, *V_appl_* was set to 1.32 V for the p‐osc‐1 operation and 1.75 V for the p‐osc‐2 operation. 5 µs pulses were applied. The Q0 output responds immediately to the magnitude of *V_appl_*, while Q1 switches from 0 to 1 at the moment when the first oscillation event occurs. In the actual operation, the pulse width can be controlled and is defined as *t_mtr_*. For example, when *t_mtr_* = 2.5 µs, the Q1 values in the four examples are 0, 1, 1, and 0, respectively. As such, the output of the p‐trit circuit can be tuned by varying both *V_appl_* and *t_mtr_*.

Figure 3d shows the experimentally obtained probability distributions of the three states. Here, the *V_appl_* ​ ranges were from 1.290 to 1.790 V, with a step size of 5 mV for the p‐osc‐1 regime (1.290–1.360 V) and the p‐osc‐2 regime (1.720–1.790 V), and 10 mV for the osc regime (1.360–1.720 V). For each *V_appl_*, 50 oscillation signals were measured, and the corresponding Q0 and Q1 outputs were recorded at *t_mtr_* = 2.5 µs. These results confirm that the proposed NbO_x_ p‐trit successfully produces three probabilistic output states with a sigmoid‐like response, achieving a bit operation rate of 2.5 µs/bit. Additionally, the NbO_x_ oscillator, which is the primary source of energy consumption, is estimated to consume 2.4 nJ/bit. The relatively small energy contribution from the peripheral CMOS circuitry is separately analyzed and benchmarked in Table S1. These results establish a practical basis for TPM hardware implementation (Note S5).

Furthermore, this probabilistic behavior can be tuned by adjusting the *t_mtr_*. Figure 3e shows the variation of the oscillation probability as a function of the *t_mtr_*, where a longer *t_mtr_* results in a wider *V_appl_* range. This tendency reflects the increased oscillation probability within a longer observation period. A shorter *t_mtr_* results in a sharper probability distribution between states (i.e., more deterministic), but offers the advantage of faster sampling and reduced energy consumption. Therefore, by balancing these trade‐offs, probabilistic computing can be optimized to achieve both faster solution search and lower energy consumption.

Since the probability distribution depends on *t_mtr_*, the effect of external noise sources, such as timing jitter, on the probability distribution should also be considered. The proposed p‐trit shows nearly identical probabilistic characteristics under a timing uncertainty of ±0.2 µs around the nominal value of *t_mtr_* = 2.5 µs, demonstrating that its probabilistic behavior is sufficiently robust against external timing noise (the robustness against timing jitter is presented in Figure S9).

### 2.4 Ternary Potts Machine Hardware Demonstration with Virtual p‐trits

To validate the system‐level feasibility of the proposed ternary probabilistic computing framework based on the experimentally characterized NbO_x_ p‐trit (hereafter simply p‐trit), we constructed a prototype TPM using MCU‐based virtual p‐trits. In the present stage, MCU‐based virtual p‐trits were employed because robust packaging and large‐scale integration technologies for the discrete NbO_x_ devices are not yet available. Nevertheless, the virtual p‐trit was designed to reflect the physical constraints and non‐idealities of the actual device, thereby providing a practical basis for future fully integrated p‐trit implementations.

**FIGURE 4:**
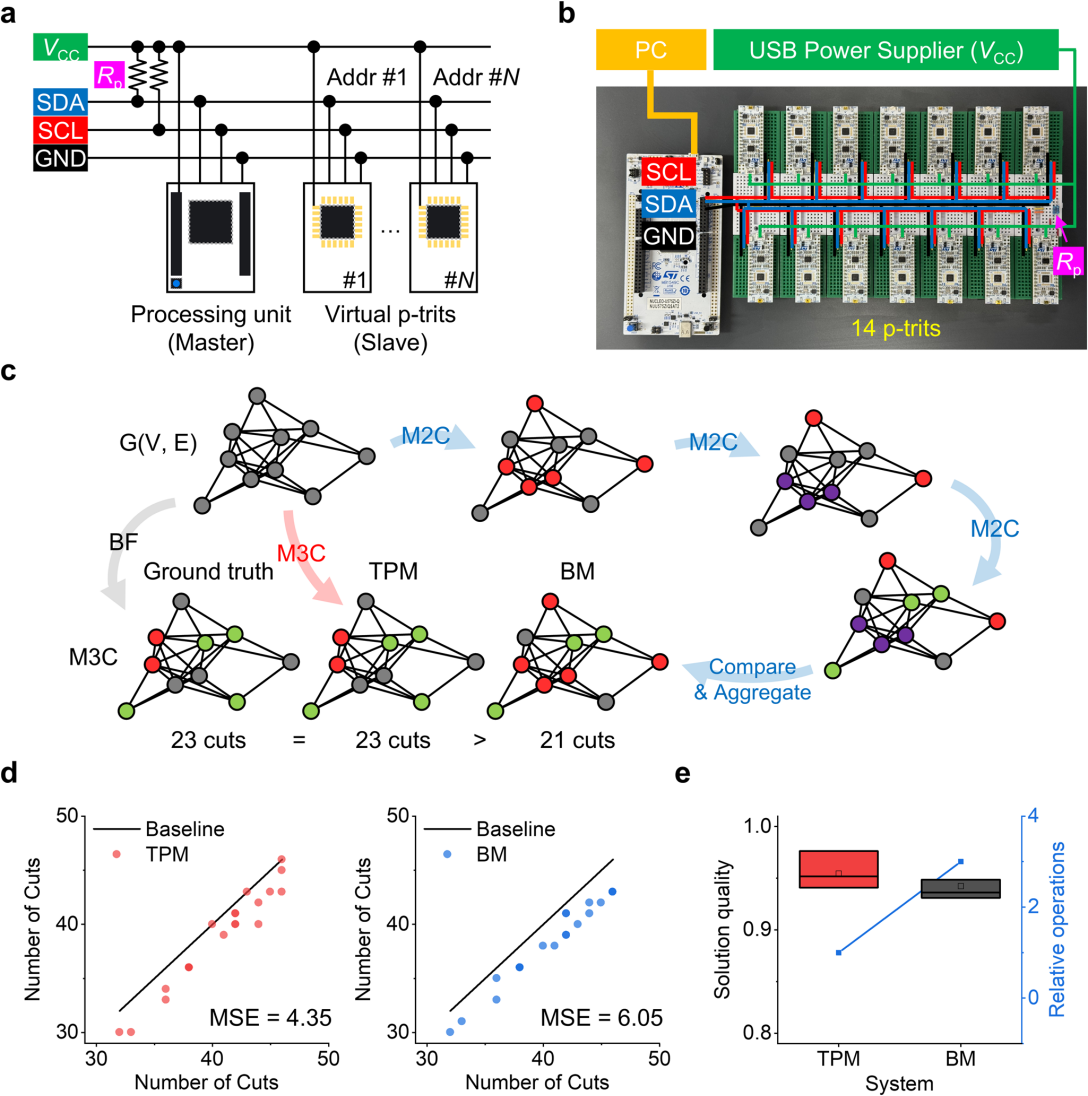
TPM and BM system demonstration implemented on an MCU‐based hardware framework. (a, b) System configuration of the MCU‐based TPM, schematic (a) and Experimental setup (b). (c) M3C problem example and its solving process and results using BF, TPM, and BM. (d) Accuracy comparison on 20 random graphs (*N* = 14). Baseline (black line) is the ground truth from BF. (e) Comparison on solution quality and relative operations.

Figure 4a illustrates the structure of the MCU‐based TPM system, where virtual p‐trits, programmed into the MCU, are connected in parallel to the processing unit. The processing unit collects the p‐trit's outputs, computes $\theta_{i}^{min}$ for each node using Equation S3.6 (Note S3), and linearly maps $\theta_{i}^{min}$ onto the *V_appl_* for the subsequent iteration. A photograph of the experimental setup is shown in Figure 4b, with colors matching the system diagram. Additional implementation details and experimental results are presented in the Experimental section and Note S6.

To benchmark system performance, we applied both p‐trit‐based TPM and p‐bit‐based BM systems to solve the M3C problem—a more complex combinatorial optimization problem than the binary Max‐2‐Cut (M2C) [40, 41]. The M3C problem aims to divide the vertices of a graph into three groups to maximize the sum of the weights of the edges connecting vertices in different groups. The energy of the max‐cut problem for a derived configuration is defined as:

(6) $$H\left(x\right)=\sum_{i<j}J_{ij}x_{i}\cdot x_{j}\;$$

where ***x**_i_* represents the state vector of node *i*, and *J_ij_* is the weight of the edge between nodes *i* and *j*. In this problem, the grouping is determined by the state of each node; therefore, in the TPM, three groups can be defined as three distinct states, making it an ideal example to demonstrate the advantages of the p‐trit.

Figure 4c shows an example of the graph problem with 10 vertices and 25 fixed‐weight edges, and the M3C solutions obtained by brute‐force (BF), the proposed TPM, and a greedy approximation using BM. As the three states can represent three different groups, a single iteration of the TPM can directly yield a 3‐partition, while BM requires three consecutive 2‐cut operations—first splitting the set into two groups, and then further bisecting each of the two groups once more—to represent the three groups. This means that the BM already requires 3 times the computational overhead compared to TPM.

Finally, to comprehensively evaluate the efficiency and performance, we defined the solution quality as the ratio of the energy obtained by the solver to the global optimal energy found by BF:

(7) $$Solution\;Quality=\frac{E_{derived\;solution}}{E_{BF}}\;$$

Additionally, TPM can achieve higher accuracy than BM. Figure 4d compares the M3C results for the TPM and BM on 20 randomly generated graphs with 14 nodes and 50% connectivity. The baseline is the ground truth obtained by BF for each problem. The results show that TPM achieves a lower mean squared error (MSE = 4.35) compared to greedy BM (MSE = 6.05), indicating its higher solution accuracy.

Figure 4e presents the trade‐off between the solution quality and the relative operations. While the BM requires significantly more operations (relative operations = 3) to approximate the solution, it yields a lower solution quality. In contrast, the TPM achieves a higher solution quality (closer to 1) with a minimal operation count (relative operations = 1). These results confirm that the TPM leverages its multi‐state expressiveness to deliver both improved performance and substantially greater efficiency in converging to optimal solutions.

### 2.5 Simulation‐Based Performance Evaluation for Large‐Scale Optimization Problems

To verify the scalability and robustness of the proposed system, we evaluated the TPM on large‐scaled weighted combinatorial optimization problems. Specifically, we focused on whether the physical constraints of the p‐trit—which operates primarily within the angular range of (0,  4π/3] while omitting the cyclic path between on and off—compromises the computational capability compared to a designed ideal model. To rigorously assess this, we benchmarked the p‐trit‐based TPM against an ideal TPM using the ternary number partitioning problem (TNPP) as a testbed.

The number partitioning problem (NPP) is a representative NP‐hard weighted combinatorial optimization problem that aims to divide a given set of numbers into subsets whose sums are as equal as possible [42, 43]. This problem is closely related to practical tasks such as load balancing, task scheduling, and resource allocation, where limited resources must be evenly distributed among multiple groups [44, 45, 46, 47]. Specifically, we used a TNPP, an extended form of the NPP that divides a set of numbers into three subsets, making it an ideal problem for evaluating the TPM. As the simplest non‐binary extension, TNPP provides a fundamental framework for multi‐class or multi‐valued problems, featuring a more complex state space and energy landscape than the binary case.

**FIGURE 5:**
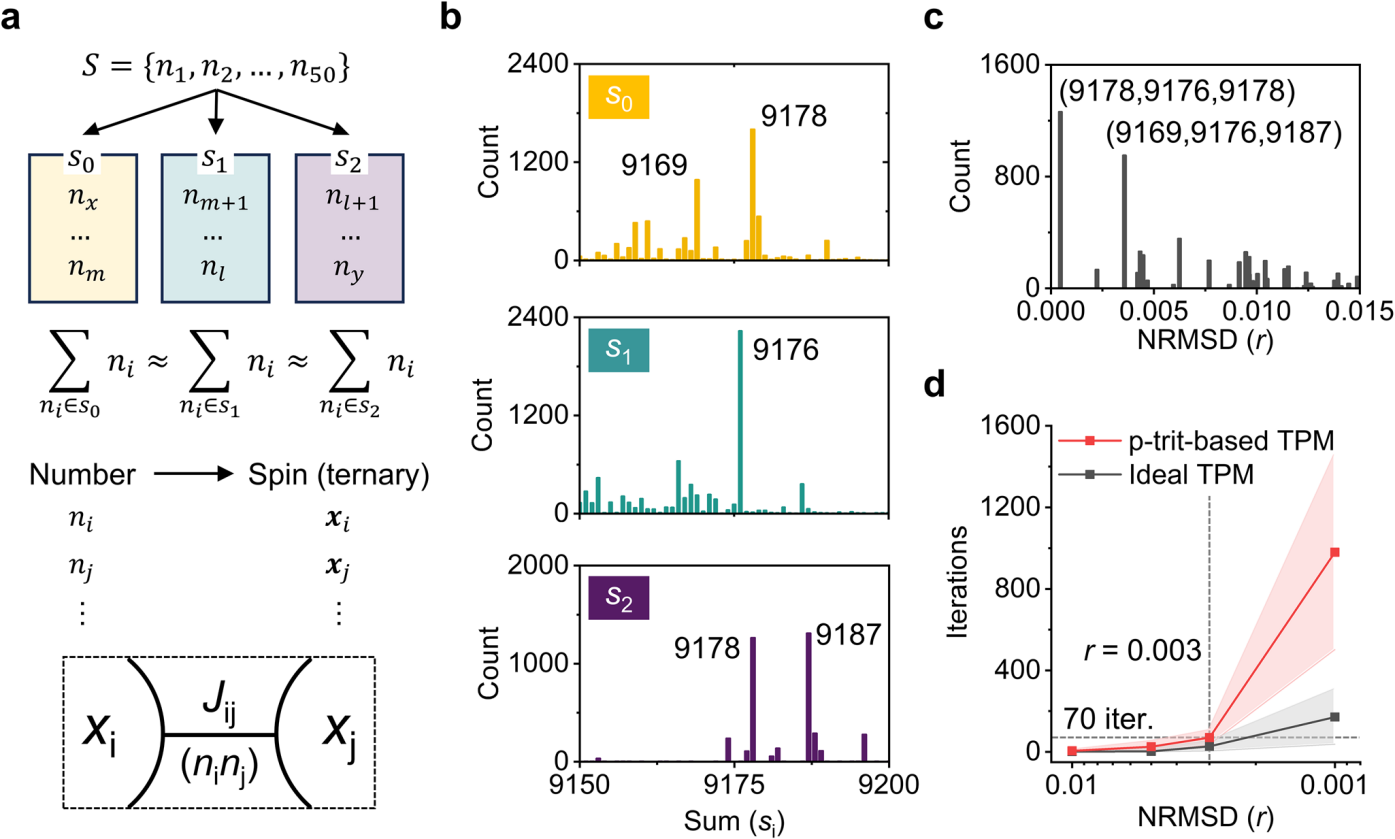
TNPP using ideal and p‐trit‐based TPMs. (a) Schematic and formulation of TNPP of 50 number set *S*. (b) Histograms of the individual subset sums of *s*_0_,*s*_1_, and *s*_2_ over 100 00 iterations. (c) Histogram of the partition solutions plotted against NRMSD (*r*). (d) Comparison of the number of iterations required to reach target NRMSD for the ideal and p‐trit‐based TPMs.

Figure 5a shows the schematic and formulation of TNPP. When a set of positive integers with a size of *N* (in this case, *N* = 50) is given, the energy is defined as follows [43]:

(8) $$H=A{\left(\sum_{i=1}^{N}n_{i}x_{i}\right)}^{2}$$

where *A* is a normalization constant, *n_i_* is the *i*‐th element of set *S*, and ***x**_i_* is a two‐dimensional unit vector defined as ***x**_i_* =  (cos θ_*i*_, sin θ_*i*_) with $\theta_{i}\in \left\{0,\frac{2\pi }{3},\frac{4\pi }{3}\right\}$. As a result, the interaction coefficient *J_ij_* between ***x**_i_* ​ and ***x**_j_* ​ in Equation (1) can be derived as *J_ij_* = *n_i_* *n_j_* (Note S7).

We solved this problem using both the ideal TPM and the p‐trit‐based TPM and evaluated the quality of the obtained solutions. The reference solutions were obtained using the Gurobi Optimizer, a classical mixed‐integer programming solver based on a branch‐and‐bound tree search algorithm [48]. Figure 5b–d show the TPM simulation results using the p‐trits for a randomly generated set *S* consisting of 50 elements, each being a positive integer between 1 and 1000 (Note S8). Figure 5b shows the histogram of the sum of the three subsets *s*_0_,*s*_1_, and *s*_2_ over 100 00 iterations. The Gurobi optimizer yields three subsets with sums $\sum_{x_{i}\in S_{k}}x_{i}\in$ {9178, 9177, 9177}.

To evaluate the solution quality independent of the problem scale, we utilized the normalized root mean square difference (NRMSD, denoted as *r*), defined as *r*  =  *RMSD*/*r*_0_. The root mean square difference (RMSD) and the normalization factor *r*_0_ are defined as follows:

(9) $$\begin{array}{ccc} & & \mathrm{RMSD}=\sqrt{\frac{{\left(s_{0}-s_{1}\right)}^{2}+{\left(s_{1}-s_{2}\right)}^{2}+{\left(s_{2}-s_{0}\right)}^{2}}{3}}\;,\\ & & r_{0}=\sqrt{\frac{2}{3}\sum_{i=1}^{N}{n_{i}}^{2}} \end{array}$$

Here, *r*_0_ is a theoretical standard deviation of the pairwise group‐sum difference in a uniformly random ternary partition (Note S9). For the specific set *S* used in this study, *r*_0_ is approximately 3601.

Figure 5c shows the histogram of the partition solutions obtained over 100 00 iterations, plotted against their corresponding NRMSD values. The distribution clearly shows that the p‐trit TPM most frequently identifies solutions in the high‐precision regime (e.g., *r* ≈ 0.0004, 99.96% reduction in error relative to *r*_0_), which corresponds to the near‐optimal partition (9178, 9176, 9178). Although this solution is not the theoretical optimum (*r* ≈ 0.0002), it is very close to it. These results demonstrate that the p‐trit‐based TPM can effectively solve the example TNPP.

Figure 5d compares the number of iterations required to reach specific NRMSD thresholds for the ideal TPM (black line) and the p‐trit‐based TPM (red line). The results represent the average required iterations of 10 trials started from random initial states. Although the p‐trit TPM requires slightly more iterations than the ideal model, it exhibits a remarkably similar convergence trajectory. Crucially, the number of iterations needed to reach the high‐precision target of *r*  = 0.003 (dashed line) remains within approximately 70, which is of the same order of magnitude as the ideal model. Collectively, these results confirm that the proposed p‐trit captures the core advantages of the ternary scheme, maintaining computational robustness comparable to the theoretical ideal even under physical device constraints, thereby validating its scalability for large‐scale combinatorial optimization. Furthermore, identical TNPP simulations performed for a larger system size (*N* = 90) also exhibited consistent scalability and robustness of the TPM, confirming the reproducibility of the observed performance trends (Table S2).

## 3 Conclusions

In this work, we introduced a physically realizable ternary probabilistic computing element (p‐trit) based on the intrinsic oscillatory properties of NbO_x_ threshold‐switching memristors. By integrating this device into a ternary Potts machine (TPM), we successfully demonstrated a compact and energy‐efficient hardware solution for solving combinatorial optimization problems. Our experimental and simulation results confirm that the proposed p‐trit‐based architecture significantly outperforms binary counterparts in both solution accuracy and computational efficiency, marking a crucial step toward scalable multinary probabilistic computing, with a compact implementation advantage over existing multinary probabilistic hardware (Table S3).

While NbO_x_ is employed in this study, any oscillator system that exhibits three distinct operating regimes arising from two successive NDR boundaries can, in principle, be used as a p‐trit, including VO_2_‐based systems [49]. Exploring a broader range of materials suitable for p‐trit operation and comparing their performance will be an interesting direction for future research. In particular, thermodynamic engineering of the device and substrate may offer a practical route to improve both operation speed and stochastic characteristics by tailoring the electrothermal dynamics of the p‐trit [25].

Looking beyond the p‐trit, the realization of multinary probabilistic computing represents an important future direction. While the search for physical devices with intrinsic multi‐state stochastic behavior should continue, such an approach remains highly challenging. We anticipate that a practical path forward will emerge from the co‐design of hardware devices and system‐level architectures. As summarized in Table S3, several approaches have been proposed to implement multinary probabilistic hardware based on conventional p‐bits, typically relying on multiple stochastic channels, device branches, comparators, or latches to represent multinary state variables [2, 11, 14, 15, 21]. By incorporating the p‐trit into these algorithmic and architectural frameworks, multinary probabilistic computing is expected to be realized with improved efficiency.

The proposed p‐trit operation is limited to an ordinal state transition pathway (off‐oscillation‐on), excluding a direct on–off transition pathway, which is required for an ideal p‐trit. This restricted operation can lead to slower convergence and larger fluctuations compared with an ideal p‐trit (Figure S11). This issue could be addressed by applying a look‐up‐table‐based remapping scheme or by implementing an n‐bit‐counter‐based circuit architecture (Figure S12). On the other hand, the ordinal nature of the p‐trit is not necessarily a critical limitation for problems involving inherently ordinal data representations (Figure S13). In summary, while the proposed p‐trit may impose constraints, its impact is problem‐dependent. Furthermore, this non‐ideality can be mitigated through circuit‐ or algorithm‐level compensation schemes, enabling the p‐trit to maintain substantial advantages over conventional p‐bits.

Another important issue associated with the proposed p‐trit is device‐to‐device and cycle‐to‐cycle uniformity. Since the p‐trit utilizes two probabilistic operating regions, it is inherently more sensitive to variation than conventional p‐bit technologies, which could complicate practical large‐scale integration. Our p‐trit also showed a cycle‐dependent shift in the probabilistic operating range. However, while the operation voltage range shifts with cycling, the operating voltage window itself remains nearly unchanged. Therefore, this drift issue is expected to be correctable using previously reported calibration schemes previously established for p‐bit systems [2, 3, 8, 10]. (See Note S10 for the cycle‐dependent operating window drift results and proposed correction strategy).

Furthermore, beyond combinatorial optimization, the proposed multinary probabilistic primitive and sampling rule provide a versatile foundation for broader applications. By bridging the gap between theoretical probabilistic models and physical device characteristics, this work paves the way for advanced computing paradigms, including statistical inference, multi‐class learning, and energy‐efficient neuromorphic computing.

## 4 Experimental Section

### 4.1 Device Fabrication

A NbO_x_ threshold switching memristor (TiN/NbO_x_/TiN‐via) was fabricated using the following process. A planarized substrate containing 150 nm‐diameter TiN vias, provided by a commercial foundry, served as the bottom electrode. For the 100 µm‐square device patterning, a mask aligner (Midas MDA‐600S) was used, with AZ 5214E and AZ 300 MIF Developer employed as the photoresist and developer, respectively. After photolithographic patterning, a 20 nm‐thick NbO_x_ blanket layer was deposited by reactive sputtering at 100°C using an Nb target under a mixed gas flow of Ar and O_2_ (Ar: O_2_ = 50:4) in a co‐sputtering system (DAE‐KI Hitech). Subsequently, a 50 nm‐thick TiN top electrode was deposited by reactive sputtering at room temperature using a TiN target. The lift‐off process was then carried out in acetone to define the TiN electrode pattern. Finally, crystallization of the NbO_x_ layer was achieved by rapid thermal annealing (RTA) at 750°C for 2 min using an RTP‐1300 system (ECOPIA).

### 4.2 Electrical Measurement

All electrical characterizations were performed using a semiconductor parameter analyzer (Keithley 4200A‐SCS) and a probe station system. The *I–V* characteristics were obtained in a current sweep mode using two Source Measurement Units (SMUs). For the self‐oscillation measurements, voltage pulses with various amplitudes were applied and measured using a Keithley 4225‐PMU (Pulse Measurement Unit) and 4225‐RPM (Remote Amplifier/Switch).

For the experimental demonstration of the NbO_x_ p‐trit with CMOS peripheral circuits, a comparator (TLV3201, Texas Instruments) and a D flip‐flop (74LVC74AD, NXP Semiconductors) were used. The DC bias was supplied using a power supply (K6135A, EXSO), and the output waveforms were monitored by a digital oscilloscope (MHO5054, RIGOL). A microcontroller unit (MCU; NUCLEO‐U575ZI‐Q, STMicroelectronics) was used to generate input voltage pulse signals and a clear pulse to the D flip‐flop.

In the MCU‐based TPM system, the NUCLEO‐U575ZI‐Q (STMicroelectronics) MCU was used as the processing unit, while STM32L432KCU6 (STMicroelectronics) MCUs were employed as virtual p‐trits, interconnected via an I^2^C (Inter‐Integrated Circuit) communication bus for data exchange and update control.

## Author Contributions

**Gwangmin Kim**: conceptualization, investigation, validation, software. **Tae Wook Go**: investigation, validation, formal analysis. **Hanchan Song**: conceptualization, investigation, methodology, software. **Younghyun Lee**: investigation, validation. **Hakseung Rhee**: conceptualization, methodology, software, data curation, investigation, validation, formal analysis, visualization, writing – original draft, writing – review and editing. **Seoeun Jang**: writing – original draft, methodology, software, data curation, investigation, validation, formal analysis, writing – review and editing. **Woojoon Park**: investigation, validation, formal analysis, software. **Junmo Kang**: investigation, validation. **Do Hoon Kim**: investigation, methodology, software. **Kyung Min Kim**: supervision, funding acquisition, writing – original draft, writing – review and editing, project administration, resources. **Daehee Kim**: investigation, validation.

## Funding

This work was supported by the National Research Foundation of Korea (NRF) (Grant numbers: RS‐2023‐00216619, RS‐2023‐NR077077, RS‐2023‐00216992, RS‐2026‐25542514, and RS‐2025‐02433006).

## Conflicts of Interest

The authors declare no conflicts of interest.

## Supporting information




**Supporting File**: advs76754‐sup‐0001‐SuppMat.docx.

## Data Availability

The data that support the findings of this study are available from the corresponding author upon reasonable request.
