# Supplementary material for: NbOx Mott Memristor‐Based Oscillatory P‐trit for Ternary Potts Machine
Source: Adv Sci (Weinh). 2026 Jul 27;13(58):e76754. doi: 10.1002/advs.76754 (PMC13403740; doi:10.1002/advs.76754)
Supplement: Supplementary file 1 [file ADVS-13-e76754-s001.docx]

Supporting Information

NbO_X_ Mott Memristor-based Oscillatory P-trit for Ternary Potts Machine

*Hakseung Rhee*†*, Seoeun Jang*†*, Tae Wook Go, Woojoon Park, Gwangmin Kim. Do Hoon Kim, Hanchan Song, Younghyun Lee, Junmo Kang, Daehee Kim, and Kyung Min Kim**

H. Rhee, T. W. Go, D. H. Kim, D. Kim, K. M. Kim

Department of Materials Science and Engineering

Korea Advanced Institute of Science and Technology (KAIST),

Daejeon 34141, Republic of Korea

S. Jang, J. Kang, K. M. Kim

Graduate School of Semiconductor Technology

Korea Advanced Institute of Science and Technology (KAIST),

Daejeon 34141, Republic of Korea.ss

W. Park

Peter-Grünberg-Institut 14 (PGI-14), Forschungszentrum Jülich GmbH,

Jülich 52425, Germany

G. Kim

Peter-Grünberg-Institut 7 (PGI-7), Forschungszentrum Jülich GmbH,

Jülich 52425, Germany

H. Song

Electronics and Telecommunications Research Institute (ETRI)

Daejeon 34129, Korea

Y. Lee

Center for Semiconductor Technology

Korea Institute of Science and Technology (KIST)

Seoul 02792, Republic of Korea

Email: km.kim@kaist.ac.kr

**Figure S1. Conceptual overview of the NbO_X_ p-trit operation and SPS rule.** (Upper right) Schematic of p-trit vector spin representation and the SPS rule. The three ternary states ($s_{0}$, $s_{1}$, $s_{2}$) are represented as unit vectors separated by $2\pi/3$on a 2D plane. For a given input $\theta_{i}$, the SPS rule confines the probabilistic transition to the two energetically neighboring states, resulting in pairwise probabilistic transitions (off↔osc and osc↔on) with sigmoidal probability profiles, while the transition to the farthest state is suppressed. (Left and bottom) Schematic of the NbO_X_ p-trit circuit comprising the NbO_X_ oscillator and FTTO-based readout, producing three ternary states (off, osc, on). Representative current waveforms at selected $V_{appl}$values within the p-osc-1 (off↔osc) and p-osc-2 (on↔osc) probabilistic regions are shown, illustrating the stochastic nature of the first oscillation event timing (FTTO). The FTTO captures the sigmoidal relationship between $P_{osc}$and $V_{appl}$, defined as the probability that the first oscillation event occurs within the monitoring window $t_{mtr}$. The sigmoidal property of the NbO_X_ p-trit shows that shorter FTTO windows correspond to the osc region, while longer FTTO windows correspond to the off and on regions.

Supplementary Note 1. Probabilistic sampling process of a Boltzmann machine

On a Boltzmann machine consisting of multiple binary probabilistic elements (p-bits) $x_{i}$ whose value can be 0 or 1, an update rule that follows a sigmoidal function of p-bit is proved as follows.

If the probability of achieving the state $x_{j}=1$ follows a Boltzmann distribution,

$$P\left( x_{j}=1 \right)=\frac{1}{Z}e^{-\frac{E(x_{j}=1)}{kT}}, Z=\sum_{All states, k} e^{-\frac{E_{k}}{kT}}$$

(S1.1)

where $E\left( \vec{x} \right)$ is an energy defined by each state of p-bits. With a slight variation of Equation S1.1,

$$\ln\left( P\left( x_{j}=1 \right) \right)=-\frac{E\left( x_{j}=1 \right)}{kT}-\ln\left( Z \right)$$

(S1.2)

$$E\left( x_{j}=1 \right)=-kT(\ln\left( P\left( x_{j}=1 \right)+\ln\left( Z \right) \right)$$

(S1.3)

By the same token, if we expand the expression for the state when $x_{j}=0$,

$$E\left( x_{j}=0 \right)=-kT(\ln\left( P\left( x_{j}=0 \right)+\ln\left( Z \right) \right)$$

(S1.4)

Then, the resulting change in $E$ when $x_{j}$ is transformed from 0 to 1 is

$$\Delta E\left( x_{j}=0\to1 \right)=E\left( x_{j}=1 \right)-E\left( x_{j}=0 \right)=-kT\left( \ln\left( P\left( x_{j}=1 \right) \right)-\ln\left( P\left( x_{j}=0 \right) \right) \right)$$

(S1.5)

With a slight variation of Equation S1.5,

$$\Delta E\left( x_{j}=0\to1 \right)=-kT\times\ln\left( \frac{P\left( x_{j}=1 \right)}{P\left( x_{j}=0 \right)} \right)=-kT\times\ln\left( \frac{P\left( x_{j}=1 \right)}{1-P\left( x_{j}=1 \right)} \right)$$

(S1.6)

$$kT\times\ln\left( \frac{1}{P\left( x_{j}=1 \right)}-1 \right)=\Delta E\left( x_{j}=0\to1 \right)$$

(S1.7)

Then, the $P\left( x_{j}=1 \right)$ is described as Equation S1.8.

$$P\left( x_{j}=1 \right)=\frac{1}{1+e^{\frac{\Delta E\left( x_{j}=0\to1 \right)}{kT}}}=\frac{1}{1+e^{\frac{\Delta E_{0\to1}}{kT}}}$$

(S1.8)

Considering that $x_{i}$ is a binary probabilistic element,

$$\Delta E_{0\to1}=\frac{E\left( x_{j}=1 \right)-E(x_{j}=0)}{1}=\frac{\partial E}{\partial x_{j}}$$

(S1.9)

Thus, if we assume a quantity $I_{j}=-\frac{\partial E}{\partial x_{j}}$, the $P\left( x_{j}=1 \right)$ is expressed as

$$P\left( x_{j}=1 \right)=\frac{1}{1+e^{-\frac{I_{j}}{kT}}}$$

(S1.10)

which follows a sigmoidal function, $y=\frac{1}{1+e^{-x}}$.

Supplementary Note 2. Segmented Probabilistic Sampling (SPS) rule of a ternary Potts machine (TPM)

The possible states of the constituent elements (ternary probabilistic unit, p-trit) of a ternary Potts machine are based on the Potts model. Specifically, a p-trit with ternary states outputs three spin states uniformly distributed along a circle. Therefore, these three spin states are defined as unit vectors in a 2D plane, where each state can only output allowed states of $0, \frac{2\pi}{3}$, and $\frac{4\pi}{3}$.

$$\boldsymbol{x}_{i}=\left( \begin{matrix} \cos\theta_{i} \\ \sin\theta_{i} \end{matrix} \right), \theta_{i}\in\left\{ 0, \frac{2\pi}{3},\frac{4\pi}{3} \right\}$$

(S2.1)

Furthermore, based on the principles of the Boltzmann machine, the Hamiltonian of a Potts machine composed of p-trits is constructed as follows. When assuming that the occurrence of each energy state follows a Boltzmann distribution, it can be described as below, and the sum of probabilities $\theta_{i}$ is being $0, \frac{2\pi}{3}$ or $\frac{4\pi}{3}$is equal to 1.

$$H=\sum_{i\neq j} J_{ij}\boldsymbol{x}_{i}\cdot\boldsymbol{x}_{j}$$

(S2.2)

$$P\left( H_{k} \right)=\frac{e^{-\frac{H_{k}}{kT}}}{Z}, Z=\sum_{n}^{N} e^{-\frac{H_{n}}{kT}}$$

(S2.3)

$$P_{\theta_{i}=0}+P_{\theta_{i}=\frac{2\pi}{3}}+P_{\theta_{i}=\frac{4\pi}{3}}=1$$

(S2.4)

The probabilistic transition from the current state $\boldsymbol{x}_{n}$ to the next state $\boldsymbol{x}_{n}^{*}$ is designed to drive the system towards energy minimization. The local Hamiltonian of the system at state $\boldsymbol{x}_{n}$ is given by $H_{n}=(\sum J_{ni}\boldsymbol{x}_{i})\cdot\boldsymbol{x}_{n}$. Here, $\boldsymbol{x}_{n}^{*}$ is the result that satisfies $\mathrm{argmin}_{\boldsymbol{x}_{n}^{*}} (H_{n})$. Therefore, considering $\boldsymbol{x}_{n}$ as a vector, $\boldsymbol{x}_{n}^{*}$ is oriented in the direction opposite to $\sum J_{ni}\boldsymbol{x}_{i}$.

In the defined ternary system, the angle $\theta_{n}$ of state $\boldsymbol{x}_{n}$ can assume only three allowed values, $0, \frac{2\pi}{3}$, and $\frac{4\pi}{3}$. Here, we define $\theta_{n}^{min}$ as the angle that is opposite to the direction of the aforementioned $\sum J_{ni}\boldsymbol{x}_{i}$.

To ensure this system ultimately adheres to a Boltzmann distribution, the acceptance probabilities from $\theta_{n}^{min}$ to each state are defined as follows, based on the Metropolis-Hastings algorithm.

$$P\left( \theta_{n}^{min}\to\theta_{n}^{*} \right)=P_{\theta_{n}^{min}\to\theta_{n}^{*}}=e^{-\frac{\Delta H_{\theta_{n}^{min}\to\theta_{n}^{*}}}{kT}}$$

(S2.5)

In this scenario, a transition to the state farthest from $\theta_{n}^{min}$ involves a significantly larger energy increase compared to transitions to the other two states. Consequently, the acceptance probability of such a transition can be said to be nearly zero. Assuming $\theta_{n}^{min}\in\left[ 0, \frac{2\pi}{3} \right)$, the farthest state is $\theta_{n}^{*}=\frac{4\pi}{3}$, transitions to the remaining two states then virtually accounts for almost all the probability. For this reason, the equation can be simplified as follows:

$$P_{\theta_{n}^{*}=0}+P_{\theta_{n}^{*}=2\pi/3}=1$$

(S2.6)

Therefore, by following the process below based on the Boltzmann distribution, we can draw the following conclusion.

$$\ln\left( P_{\theta_{n}=0} \right)=-\frac{H_{\theta_{n}=0}}{kT}-\ln\left( Z \right), \ln\left( P_{\theta_{n}=2\pi/3} \right)=-\frac{H_{\theta_{n}=2\pi/3}}{kT}-\ln\left( Z \right)$$

(S2.7)

$$\ln\left( P_{\theta_{n}=2\pi/3} \right)-\ln\left( P_{\theta_{n}=0} \right)=\ln\left( \frac{P_{\theta_{n}=2\pi/3}}{P_{\theta_{n}=0}} \right)=-\frac{H_{\theta_{n}=2\pi/3}-H_{\theta_{n}=0}}{kT}$$

(S2.8)

$$\ln\left( \frac{1}{P_{\theta_{n}=2\pi/3}}-1 \right)=\frac{H_{\theta_{n}=2\pi/3}-H_{\theta_{n}=0}}{kT}$$

(S2.9)

$$\frac{1}{P_{\theta_{n}=2\pi/3}}=1+e^{\frac{H_{\theta_{n}=2\pi/3}-H_{\theta_{n}=0}}{kT}}$$

(S2.10)

$$\therefore P_{\theta_{n}=2\pi/3}=\frac{1}{1+e^{-\frac{I_{n}}{k^{'}T}}},I_{n}=-\frac{\partial H}{\partial\theta_{n}}, I_{n}\in\left[ 0, \frac{2\pi}{3} \right), k^{'}=\frac{3k}{2\pi}$$

(S2.11)

By cyclic symmetry, the same probabilistic form applies to all three angular intervals:

$$P_{\theta_{i}=2n\pi/3}=\frac{1}{1+e^{-\frac{I_{i}}{k^{'}T}}}, I_{i}\in\left[ \frac{2\left( n-1 \right)\pi}{3}, \frac{2n\pi}{3} \right), n\in\{1, 2, 3\}$$

(S2.12)

Importantly, the ternary nature of the p-trit is defined by the three equivalent vector spin states separated by $2\pi/3$ in the Potts representation, rather than by simultaneous accessibility of all three transition paths under every local field condition. Within each interval, the SPS rule derived from the Boltzmann distribution allows only the two neighboring states to possess a nonzero probability, whereas the third, farthest state is excluded due to its prohibitively high energy. For example, in the interval $\left[ 0, \frac{2\pi}{3} \right)$, $P_{\theta_{i}=2\pi/3}$ is given by Equation S2.12, while the other two states satisfy $P_{\theta_{i}=0}=1-P_{\theta_{i}=2\pi/3}$ and $P_{\theta_{i}=4\pi/3}=0$.

Supplementary Note 3. Energy minimization process of TPM

The local Hamiltonian of a node $\boldsymbol{x}_{n}$ is given by

$$H_{n}=\left( \sum J_{ni}\boldsymbol{x}_{i} \right)\cdot\boldsymbol{x}_{n}\boldsymbol{=}\sum_{j\neq n}^{N} J_{nj}\cos(\theta_{j}-\theta_{n})\boldsymbol{=}\left( \sum_{j\neq n}^{N} J_{nj}\cos\theta_{j} \right)\cos\theta_{n}+\left( \sum_{j\neq n}^{N} J_{nj}\sin\theta_{j} \right)\sin\theta_{n}$$

(S3.1)

The two summation terms represent the collective influence of the neighboring spins projected on the real and imaginary axes of a two-dimensional spin plane. This collective influence can be expressed as a local effective field $\boldsymbol{h}_{n}$ acting on $\boldsymbol{x}_{n}$**:**

$$\boldsymbol{h}_{n}=\binom{\sum_{j\neq n}^{N} J_{nj}\cos\theta_{j}}{\sum_{j\neq n}^{N} J_{nj}\sin\theta_{j}}$$

(S3.2)

Hence, $H_{n}=\boldsymbol{h}_{n}\cdot\boldsymbol{x}_{n}$.The magnitude of $\boldsymbol{h}_{n}$ represents the total coupling strength applied to $\boldsymbol{x}_{n}$, and its direction gives the collective orientation of the surrounding spins.

The energy $H_{n}$ is minimized when $\boldsymbol{x}_{n}$ is oriented in the direction opposite to $\boldsymbol{h}_{n}$. Therefore, the optimal orientation $\theta_{n}^{min}$ that minimizes $H_{n}$ can be found as the direction opposite to the argument of $\boldsymbol{h}_{n}$:

$$\theta_{n}^{min}=\arg(\boldsymbol{h}_{n})+\pi$$

(S3.3)

To evaluate this argument, the complex representation of the local field is in,

$$z_{nb}=A+Bi=\sum_{j\neq n}^{N} J_{nj}\cos\theta_{j}+i\sum_{j\neq n}^{N} J_{nj}\sin\theta_{j}$$

(S3.4)

The argument (phase angle) of $z_{nb}$ gives the collective orientation of the neighboring spins,

$$\arg\left( z_{nb} \right)=\tan^{-1} \left( \frac{\sum_{j\neq n}^{N} J_{nj}\sin\theta_{j}}{\sum_{j\neq n}^{N} J_{nj}\cos\theta_{j}} \right)$$

(S3.5)

Thus, the minimizing direction $\theta_{n}^{min}$ of node $\boldsymbol{x}_{n}$ is

$$\theta_{n}^{min}=\arg\left( z_{nb} \right)+\pi=\tan^{-1} \left( \frac{\sum_{j\neq n}^{N} J_{nj}\sin\theta_{j}}{\sum_{j\neq n}^{N} J_{nj}\cos\theta_{j}} \right)+\pi$$

(S3.6)

In this configuration, $\boldsymbol{x}_{n}$ opposes the net influence of its neighbors, yielding $\cos(\theta_{j}-\theta_{n})=-1$ for the dominant coupling and thereby minimizing the local energy $H_{n}$ .

**Figure S2. Parasitic capacitance in the measurement setup.** Equivalent circuit diagram of the NbO_X_ p-trit measurement system, showing the intrinsic device capacitance ($\boldsymbol{C}_{\boldsymbol{M}}$ ≈ 6 pF) and the dominant parasitic capacitance of the measurement setup ($\boldsymbol{C}_{\boldsymbol{para}}$ ≈ 180 pF, contributed by probe station cables and connectors). The dominant $\boldsymbol{C}_{\boldsymbol{para}}$ acts as the effective parallel capacitance of the Pearson–Anson oscillator in the current discrete measurement setup.

**Figure S3. Effect of load resistance** $\boldsymbol{R}_{\boldsymbol{L}}$ **on the probabilistic operating window widths of p-osc-1 and p-osc-2.** (a) Current–voltage (I-V) characteristics of the NbO_X_ TSM used in this study, showing two NDR regions. (b) Oscillation probability $P_{osc}$ as a function of $\boldsymbol{V}_{\boldsymbol{appl}}$ for $R_{L}$= 500 Ω and 1 kΩ, showing the full probabilistic operating range. (c) Enlarged views of the p-osc-1 and p-osc-2 regions for each $R_{L}$ condition, with the probabilistic window width $\boldsymbol{\Delta V}_{\boldsymbol{appl}}$ indicated. At $R_{L}$ = 500 Ω, both p-osc-1 and p-osc-2 exhibit comparable window widths (~0.08 V), whereas at $R_{L}$ = 1 kΩ, the p-osc-1 window narrows to ~0.02 V and the p-osc-2 window widens to ~0.16 V, resulting in significant asymmetry.

**Figure S4. Effect of parasitic capacitance on noise-induced stochastic oscillation behavior.** Electrothermal noise simulation results of the NbO_X_ oscillator at a fixed $V_{appl}$ within the p-osc-1 probabilistic operating region, for $C_{para}$ = 50 pF (left) and $C_{para}$ = 180 pF (right). With $C_{para}$ = 50 pF, noise-induced oscillation events are not observed over the 50 µs observation window. In contrast, with $C_{para}$ = 180 pF, stochastic oscillation spikes occur with appreciable probability, demonstrating that a sufficiently large parallel capacitance is related to probabilistic operation of the NbO_X_ p-trit in the current measurement platform.

Supplementary Note 4. Oscillation speed of NbO_X_ oscillator as a function of input voltage

The oscillation frequency of the NbO_X_-based threshold-switching oscillator strongly depends on the applied bias voltage, even within the stable oscillation region. This behavior originates from the Pearson–Anson effect [1–3], where the charging and discharging dynamics of the parasitic capacitance vary nonlinearly with the applied voltage.

The oscillation period $T$can be expressed as follows:

$$T=C_{p}\left( R_{R_{L}||R_{on}}\ln\left( \frac{V_{th}-\alpha_{on}V_{appl}}{V_{h}-\alpha_{on}V_{appl}} \right)+R_{R_{L}||R_{off}}\ln\left( \frac{V_{h}-\alpha_{off}V_{appl}}{V_{th}-\alpha_{off}V_{appl}} \right) \right)$$

(S4.1)

where $\alpha_{on}=\frac{R_{on}}{R_{on}+R_{L}}$ and $\alpha_{off}=\frac{R_{off}}{R_{off}+R_{L}}$.

As indicated by Equation S4.1, the oscillation period $T$decreases monotonically with increasing $V_{appl}$, reflecting the accelerated charge-discharge transitions of the capacitor at higher bias levels.

**Figure S5. Experimental oscillation behavior of the NbO_X_ oscillator in the periodic region.** (a) Time-domain current waveforms measured at three applied voltages ($V_{appl}$ = 1.4, 1.5, and 1.6 V), each aligned to a reference peak, showing a voltage-dependent shift in oscillation period. (b) Extracted oscillation period $T$ as a function of $V_{appl}$, quantitatively confirming the dependence predicted by Equation 4.1. The continuous variation of $T$ with bias highlights the difficulty of precise peak detection in conventional counting-based probability extraction.

Experimentally, this dependence is clearly observed in Figure S1a, which presents time-domain current responses of the NbO_X_ oscillator under three different bias conditions (1.4 V, 1.5 V, and 1.6 V). Each waveform is aligned with respect to a reference peak to highlight the relative phase shifts. The subsequent peaks gradually advance as $V_{appl}$ increases, demonstrating the continuous reduction in oscillation period. Figure S1b quantitatively plots the extracted period as a function of $V_{appl}$, in agreement with the theoretical trend predicted by Equation S4.1.

This voltage-dependent variation in oscillation period makes precise peak detection and timing calibration challenging, especially near the oscillation-to-non-oscillation boundaries, where the waveform becomes more sensitive to small fluctuations in bias or temperature. Consequently, the peak-counting-based detection method requires careful adjustment of observation windows and threshold conditions to ensure reliable operation across varying input voltages.

| **Component** | **Estimation basis** | **Energy per operation** |
| --- | --- | --- |
| TLV3202 comparator *2 | $I_{Q}=40 \mu A, V_{CC}=3.3 V, t_{mtr}=2.5 \mu s$ | ~ 0.66 nJ |
| 74LVC74AD D flip-flop | $C_{PD}=19.1 pF, V_{CC}=3.3V, f=400 kHz$ | ~ 0.21 nJ |
| **Total peripheral** | **-** | **~ 0.87 nJ** |

**Table S1. Estimated energy consumption of peripheral CMOS circuitry per p-trit operation.**

Energy was estimated based on the datasheet specifications of the components used in the current discrete implementation. For the TLV3201 comparator, the quiescent current $I_{Q}$ (the static current drawn from the supply under no-load conditions) was used: $E=I_{Q}\cdot V_{CC}\cdot t_{mtr}$ , where $V_{CC}$ is the supply voltage (3.3 V) and $t_{mtr}$ is the monitoring time window (2.5 µs).

For the 74LVC74AD D flip-flop, the power dissipation capacitance $C_{PD}$ (an equivalent capacitance characterizing the dynamic power consumption due to internal switching activity) was used: $E=C_{PD}\cdot V_{CC}\cdot f$, where $f=1/t_{mtr}=400 kHz$ is the effective operating frequency.

Supplementary Note 5. Estimation of average energy consumption per bit in NbO_X_ p-trit

Based on the experimentally derived probabilistic ternary oscillation characteristics, the average energy consumption per bit generation of the NbO_X_ p-trit was quantitatively estimated. In a system where the oscillation probability varies with the applied voltage ($V_{appl}$), a comprehensive estimation of energy per bit requires a statistical model that accounts for the stochastic rate of oscillation events.

**Figure S6. Experimental FTTO distributions in the p-osc-1 region of the NbO_X_ oscillator.** Measured histogram of the FTTO under applied voltages in the p-osc-1 region ($V_{appl}$ = 1.33 V and 1.34 V). The distributions follow an exponential trend described by $f\left( t \right)=\lambda e^{-\lambda t}$, where $\lambda$ represents the mean oscillation occurrence rate.

**Figure S7. Experimental FTTO distributions in the p-osc-2 region of the NbO_X_ oscillator.** Measured histogram of the FTTO under applied voltages in the p-osc-2 region ($V_{appl}$ = 1.755 V and 1.76 V), following an exponential trend described by $f\left( t \right)=\lambda e^{-\lambda t}$.

To this end, a parameter $\lambda(V_{appl})$, which represents the mean oscillation occurrence rate as a function of the applied voltage, was introduced. The experimentally obtained FTTO distributions in the p-osc-1 (Figure S2) and p-osc-2 (Figure S3) regions, both exhibiting an exponential trend that follows the exponential distribution

$$f\left( t \right)=\lambda e^{-\lambda t}$$

(S5.1)

Here, $f\left( t \right)$ represents the probability density function of the waiting time $t$ until the first oscillation event occurs, $\lambda$ denotes the mean occurrence rate. This behavior indicates that the time until the first oscillation event obeys an exponential distribution, implying that the oscillation process can be described as a Poisson process with a constant $\lambda$. In other words, the occurrence of the first oscillation is statistically independent of the waiting time, occurring randomly with a $\lambda(V_{appl})$.

In the p-osc-1 region, each oscillation consumes approximately 102.48 pJ over a duration of 300 ns, as measured from the experimental current–voltage dynamics of the NbO_X_ oscillator. Because the oscillation intervals exhibit irregular, Poisson-like randomness (as shown in Figure 2c in main text), the expected number of oscillations within $t_{mtr}$ can be expressed as

$$N_{osc}=\lambda\left( V_{appl} \right)\cdot t_{mtr}$$

(S5.2)

The average energy consumed during $t_{mtr}$ for each $V_{appl}$ can then be obtained by multiplying $N_{osc}$ by the energy consumed per oscillation $E_{osc}$, as follows:

$$E_{avg}\left( V_{appl},t_{mtr} \right)=N_{osc}\cdot E_{osc}$$

(S5.3)

In the p-osc-1 region, the non-oscillating cases were neglected in the calculation since the device current remained nearly zero when no oscillation occurred.

In the p-osc-2 region, when oscillation occurs, each oscillation consumes approximately 140.17 pJ over a 105 ns duration, whereas in the absence of oscillation, the device remains in an on-state consuming 228.65 pJ during the same interval. However, it should be noted that the device exhibits distinct behavior. Unlike p-osc-1, the p-osc-2 region shows a burst-type oscillation pattern, in which once the first oscillation occurs, subsequent oscillations persist continuously. Therefore, in this region, the mean energy consumption was derived by calculating both the oscillation probability and the non-oscillation probability based on the experimentally extracted $\lambda\left( V_{appl} \right)$, and then estimating the expected oscillation duration during $t_{mtr}$. The resulting average energy per bit can be expressed as:

$$E_{avg}\left( V_{appl},t_{mtr} \right)=P_{osc}\cdot E_{osc}+(1-P_{osc}) \cdot E_{non-osc}$$

(S5.4)

where $P_{osc}=1-e^{-\lambda\left( V_{appl} \right)\cdot t_{mtr}}$ represents the probability of oscillation within the monitoring window, and $E_{non-osc}$ is the energy consumption during non-oscillation.

**Figure S8. Extracted mean oscillation rate** $\boldsymbol{\lambda(}\boldsymbol{V}_{\boldsymbol{appl}}\boldsymbol{)}$ **of the NbO_X_ p-trit.** Plot of the experimentally derived oscillation occurrence rate $\lambda$ as a function of applied voltage for both probabilistic regions (p-osc-1 and p-osc-2) within the monitoring time of $t_{mtr}$ = 2.5 μs.

Figure S4 plots the extracted $\lambda\left( V_{appl} \right)$ for both p-osc-1 and p-osc-2 regions. The calculated results show that, within the monitoring window of $t_{mtr}=2.5$ μs, the average energy consumption amounts to 0.242 nJ in the p-osc-1 region and 4.63 nJ in the p-osc-2 region. This difference originates from the fact that in p-osc-1, the device remains mostly in the low-power off state when oscillation does not occur, while in p-osc-2, the device stays in the high-conductance on state and continues to dissipate power even without oscillation. Averaging across the entire probabilistic operating range, the NbO_X_ device of NbO_X_ p-trit consumes approximately 2.43 nJ per bit at $t_{mtr}=2.5 \mu s$, providing a realistic estimate of the energy cost for ternary probabilistic operations.

**Figure S9. Robustness of the oscillation probability profile against** $\boldsymbol{t}_{\boldsymbol{mtr}}$ **variation.** Oscillation probability $P(t_{mtr})$as a function of applied voltage $V_{appl}$ measured at five different monitoring times ($t_{mtr}$ = 2.3, 2.4, 2.5, 2.6, and 2.7 µs). The sigmoid profiles for both the p-osc-1 (1.29–1.36 V) and p-osc-2 (1.73–1.79 V) probabilistic regions remain nearly identical across the entire variation range, confirming that the $V_{appl}\to P_{osc}$ mapping is robust against $t_{mtr}$ timing variations of ±0.2 µs (8% relative to the nominal value of 2.5 µs).

Supplementary Note 6. Experimental demonstration of MCU-based TPM operation

**Figure S10. Experimental setup and operation results of the MCU-based TPM system.** (a) Photograph of the experimental setup showing the MCU-based TPM prototype. (b) Logic analyzer capture of the I^2^C communication during the TPM operation. (c) Experimentally measured results of cuts (best_cut) and occurrence (best_freq).

An MCU-based TPM prototype was implemented using I²C communication between the processing unit (NUCLEO-U575ZI-Q, STMicroelectronics) and virtual p-trits (STM32L432KCU6). The system operation was monitored using a logic analyzer (Saleae Logic Pro 8), and the resulting cuts (best_cut) and occurrences (best_freq) were obtained from experimentally measured p-trit outputs.

Supplementary Note 7. Derivation of the interaction coefficient ($\boldsymbol{J}$) for the ternary number partitioning problem (TNPP)

The Hamiltonian for the number partitioning problem, as defined in Equation 8 of the main text, is given by:

$$H=A\left( \sum_{i=1}^{N} n_{i}\boldsymbol{x}_{i} \right)^{2}$$

(S7.1)

where $A$ is a normalization constant, $n_{i}$ is the $i$-th element of set $S$, and $\boldsymbol{x}_{i}$ is a two-dimensional unit vector defined as $\boldsymbol{x}_{i}=(\cos\theta_{i},\sin\theta_{i})$ with $\theta_{i}\in\left\{ 0,\frac{2\pi}{3},\frac{4\pi}{3} \right\}$. Expanding the square of the summation yields the following equation:

$$H=A\sum_{i=1}^{N} n_{i}n_{j}\boldsymbol{x}_{i}\boldsymbol{x}_{j}$$

(S7.2)

This double summation can be separated into self-interaction terms ($i=j$) and cross-interaction terms ($i\neq j$):

$$H=A\left( \sum_{i=1}^{N} n_{i}^{2}\boldsymbol{x}_{i}\boldsymbol{x}_{i}+\sum_{i\neq j}^{N} n_{i}n_{j}\boldsymbol{x}_{i}\cdot\boldsymbol{x}_{j} \right)$$

(S7.3)

Since $\boldsymbol{x}_{i}$ is a unit vector, the dot product of a vector with itself is unity. Consequently, the first term, $\sum_{i=1}^{N} n_{i}^{2}$, depends only on the given set of numbers and remains constant regardless of the configuration of $\boldsymbol{x}$. Because a constant offset does not affect the optimization landscape or the ground state solution, it can be omitted from the effective energy function. Focusing on the interaction terms where $i<j$, the relevant part of the Hamiltonian becomes:

$$H_{eff}\propto\sum_{i<j}^{N} n_{i}n_{j}\boldsymbol{x}_{i}\cdot\boldsymbol{x}_{j}$$

(S7.4)

Now, we compare this with the standard definition of the Ising Hamiltonian (Equation 1 in the main text):

$$H(\boldsymbol{x})=-\sum_{i<j} J_{ij}\text{ }\boldsymbol{x}_{i}\cdot\boldsymbol{x}_{j}-\sum_{i} \boldsymbol{h}_{i}\boldsymbol{x}_{i}$$

(S7.5)

In the number partitioning problem, the external field bias $\boldsymbol{h}_{i}$ is zero. By mapping the coefficients of the cross-terms in Equation S7.4 to the interaction term in Equation S7.5, the interaction coefficient $J_{ij}$ between nodes $i$ and $j$ is derived as:

$$J_{ij}=n_{i}n_{j}$$

(S7.6)

Thus, the weight of the connection between any two spins in the Ising model formulation of the TNPP is determined by the product of the corresponding numbers in the set.

Supplementary Note 8. Random integer sets used for the ternary number partitioning problem

The integer set used is listed as follows:

[865, 395, 777, 912, 431, 42, 266, 989, 524, 498, 415, 941, 803, 850, 311, 992, 489, 367, 598, 914, 930, 224, 517, 143, 289, 144, 774, 98, 634, 819, 257, 932, 546, 723, 830, 617, 924, 151, 318, 102, 748, 76, 921, 871, 701, 339, 484, 574, 104, 363]

Supplementary Note 9. Derivation of the theoretical standard deviation for random ternary partitions

Consider a set of positive numbers $\{n_{1},n_{2},\ldots,n_{N}\}$, and each element $n_{i}$ is independently assigned to one of three groups, $s_{0},s_{1}$, or $s_{2}$, with equal probability $1/3$.

Let the sum of elements in each group be

$$s_{1}=\sum_{i=1}^{N} n_{i}I_{i,1}, s_{2}=\sum_{i=1}^{N} n_{i}I_{i,2}$$

(S9.1)

where $I_{i,1}$ and $I_{i,2}$ are indicator variables that take the value 1 if $n_{i}$ belongs to group $s_{1}$ or $s_{2}$, respectively, and 0 otherwise. The difference between the sums of groups $s_{1}$ and $s_{2}$ is then

$$D_{12}=s_{1}-s_{2}=\sum_{i=1}^{N} n_{i}\left( I_{i,1}-I_{i,2} \right)=\sum_{i=1}^{N} n_{i}X_{i}$$

(S9.2)

where $X_{i}=n_{i}\left( I_{i,1}-I_{i,2} \right)$. Then, each element $n_{i}$ can contribute to $D_{12}$ in one of three ways:

$$X_{i}=\left\{ \begin{aligned} +n_{i}, if n_{i}\in s_{1} \\ -n_{i}, if n_{i}\in s_{2} \\ 0, if n_{i}\in s_{0} \end{aligned} \right.$$

(S9.3)

The expected value of $X_{i}$, $\mathbb{E}\left[ X_{i} \right]$ is zero, and $\mathbb{E}\left[ X_{i}^{2} \right]=\frac{2}{3}n_{i}^{2}$. Thus, $\mathbb{V}\left[ X_{i} \right]=\frac{2}{3}n_{i}^{2}$.

Due to the independence among the assignments of different $n_{i}$, the variance $\mathbb{V}\left[ D_{12} \right]$ and standard deviation $\sigma\left[ D_{12} \right]$ is derived as

$$\mathbb{V}\left[ D_{12} \right]\mathbb{=V}\left[ \sum_{i=1}^{N} X_{i} \right]=\sum_{i=1}^{N} \mathbb{V}\left[ X_{i} \right]=\frac{2}{3}\sum_{i=1}^{N} n_{i}^{2}$$

(S9.4)

$$\sigma\left[ D_{12} \right]=\sqrt{\frac{2}{3}\sum_{i=1}^{N} n_{i}^{2}}$$

(S9.5)

| **N** | **Ideal NRMSD (*r*)** | **NbO_X_ NRMSD (*r*)** |
| --- | --- | --- |
| 50 | 0.0029 ± 0.0016 | 0.0040 ± 0.0022 |
| 90 | 0.0019 ± 0.0015 | 0.0028 ± 0.0023 |

**Table S2. TNPP scalability evaluation across problem sizes.**

NRMSD (normalized root mean square difference, *r*) values (mean ± standard deviation) for the ideal TPM and NbO_X_ p-trit-based TPM on TNPP benchmark sets of varying scales. For each problem size N, 20 independently generated random integer sets were used (integers uniquely sampled without replacement from 1 to 20N). Each simulation was run with R = 10 independent trials per instance and 2000 iterations per trial. The most frequently appearing configuration across all trials was selected as the solution.

| **Work** | **Stochastic device** | **Stochasticity** | **Multinary rep.** | **Circuitry per node** | **State encoding** | | **Sampling overhead** |
| --- | --- | --- | --- | --- | --- | --- | --- |
| This work | NbOₓ  oscillator | Intrinsic | 3 | 1 NbOx oscillator + readout circuit | | FTTO-based p-osc regions | No |
| Borders (2019) [4] | sMTJ | Intrinsic | 2 | 1 sMTJ + readout circuit | | Binary resistance state | No |
| Whitehead (2023) [5] | SPAD | Intrinsic | q (~12) | q VRSPAD channels + q state latch | | One‑hot latch | Waiting depend on event rate |
| Cheong (2026) [6] | FB‑MOSFET | Intrinsic | q (~10) | q FB-MOSFET + q comparators | | One‑hot vector | q-dependent rejection overhead |
| Mekawy (2025) [7] | PFD-3 | Intrinsic | 3 | 1 oscillator + 1 phase readout circuit | | Analog phase | Finite settling time |
| Duffee (2025) [8] | ASIC | Extrinsic (PRNG‑based) | q (~6) | 1 digital P-dit element | | Digital isotropic p-dit | No |

**Table S3. Comparison of multinary probabilistic computing hardware systems.**

To position the proposed ternary probabilistic Mott unit within multinary probabilistic-computing hardware, we compare it with representative binary, Potts, phase-based, optical/SPAD-based, and CMOS p-dit implementations**.** The comparison highlights that many Potts or multilevel probabilistic hardware schemes require multiple devices and additional circuitry to represent a q-state variable. In contrast, the proposed TPM generates ternary stochasticity intrinsically from a single NbO_X_ Mott oscillator. This intrinsic ternary-state encoding also provides an advantage in sampling overhead. In the proposed TPM, the stochastic state is determined by first-time-to-oscillate dynamics within predefined p-osc regions, rather than by waiting for a valid event among multiple stochastic channels or rejecting invalid samples. Therefore, the output state can be obtained within a fixed measurement window, enabling self-clocked sampling without q-dependent rejection or event-waiting latency.

**Figure S11. Convergence comparison between ideal (cyclic) and NbO_X_ (ordinal) p-trit under simulated annealing.**

Convergence behavior of the ideal p-trit (cyclic, gray) and NbO_X_ p-trit (ordinal, red) on the TNPP problem with N = 50, plotted as RMS versus iteration number. The inverse temperature $\beta$was linearly increased from 0 to 0.2 over 4000 iterations. The NbO_X_ p-trit exhibits larger RMS fluctuations and slower convergence compared to the ideal cyclic model, consistent with the detour effect arising from the physically inaccessible $4\pi/3$→ $0$direct transition. Nevertheless, both models converge to near-zero RMS values as $\beta$increases, demonstrating that the ordinal constraint does not prevent convergence but affects the convergence rate.

**Figure S12. Counter-based cyclic p-trit implementation using NbO_X_ oscillator.** (a) Schematic of the counter-based cyclic p-trit circuit. (b) Operating principle of the N-bit counter-based cyclic transition via counter overflow. (c) Experimental average oscillation count as a function of $V_{appl}$, with ternary state boundaries and counter limit indicated.

The NbO_X_ oscillator serves as a stochastic random number source, generating a probabilistic number of oscillations within a fixed sampling window. Since the number of oscillations at each voltage follows a distribution, the state assignment is inherently probabilistic. An N-bit counter counts the number of oscillations, and a comparator assigns one of three ternary states ($s_{0}$, $s_{1}$, $s_{2}$) based on predefined count thresholds. As a representative example in (a), when 7 oscillations are detected, and the state boundaries are set at counts of 0, 5, and 10, the output state is determined as $s_{1}$.

As shown in (b), when the oscillation count exceeds the predefined counter limit, the counter resets, and the output wraps around from $s_{2}$back to $s_{0}$, realizing the physically inaccessible $4\pi/3$→ $0$cyclic transition. Panel (c) shows the experimental average oscillation count as a function of $V_{appl}$, confirming that the counter-based approach naturally maps onto the sigmoidal oscillation behavior of the NbO_X_ oscillator.

This approach can be generalized to other stochastic oscillator devices. However, it requires an additional N-bit counter and N-bit comparator, resulting in significant circuit area overhead compared to the FTTO-based implementation proposed in this work.

**Figure S13. MNIST digit reconstruction using RBM and TRBM.** (a) Schematic of the RBM/TRBM architecture and the NbO_X_ p-trit mapping table used in the TRBM. (b) Representative reconstruction results for original MNIST digits, RBM, and TRBM. (c) Reconstruction MSE comparison between RBM and TRBM. (d) Structural similarity (SSIM) comparison between RBM and TRBM. (e) Reconstruction MSE as a function of the number of hidden nodes for RBM and TRBM, showing faster convergence of the TRBM.

Supplementary Note 10. Stochastic-window endurance and calibration

**Figure S14. Stochastic-window endurance and calibration.** (a) Initial sigmoid fit and endurance measurement results for the p-osc-1 region ($V_{appl}$ = 1.365 V, 1.370 V, 1.375 V; $t_{mtr}$ = 2.5 μs) (b) Initial sigmoid fit and endurance measurement results for the p-osc-2 region ($V_{appl}$ = 2.05 V, 2.06 V, 2.07 V, 2.08 V; $t_{mtr}$ = 2.5 μs) (c) Extracted $V_{O}$-drift trajectory for the p-osc-1 data in (a), with $\beta$ fixed at its initial value ($\beta=146$), and the sliding-reference calibration range with a tolerance of ±2.5 mV. (d) Extracted $V_{O}$-drift trajectory for the p-osc-2 data in (b), with $\beta$ fixed at its initial value ($\beta=-110$), and the sliding-reference calibration range with a tolerance of ±2.5 mV.

The stochastic operating window of the NbO_X_ oscillator-based p-trit ranges from tens to hundreds of mV depending on the series-resistance magnitude, and the drift of the operating bias induced by repeated cycling can compromise the stability of the computing operation. To quantify this, we performed stochastic-window endurance experiments in which pulses were continuously applied at selected $V_{appl}$ values within the stochastic operating region, while the cumulative switching probability $P\left( V, t \right)=\frac{1}{1+e^{-\beta(V-V_{O})}}$ was recorded at every cycle. From the measured $P\left( V, t \right)$ data, we back-extracted $V_{O}$ at each cycle to quantify the drift tolerance and the required calibration scheme.

The stochastic operating windows of p-osc-1 and p-osc-2 drift on the mV scale under repeated cycling, with the worst case showing a drift envelope of approximately 20 mV over $2.5\times{10}^{5}$ pulses in p-osc-2. Importantly, the sigmoid steepness $\beta$ remains at its initial value throughout the entire endurance range. That is, only the half-probability point $V_{O}$ drifts, and the unified $V_{appl}\to P_{osc}$ mapping rule established in the main text therefore remains structurally valid.

Using a simple sliding-reference periodic-calibration scheme with a ±2.5 mV tolerance, the operating-point error can be bounded within half of the 5 mV step. This scheme requires only about $10-{10}^{2}$ calibration events per ${10}^{6}$ pulses, corresponding to an overhead well below 0.01%. We thus demonstrate that the cycle-induced drift of the probabilistic window is not a fundamental obstacle to large-scale implementation, but rather a bounded, slow, and correctable feature.

**References**

[1]	S. O. Pearson and H. S. G. Anson, The Neon Tube as a Means of Producing Intermittent Currents, Proceedings of the Physical Society of London **34**, 204 (1921).

[2]	G. Kim, J. H. In, Y. S. Kim, H. Rhee, W. Park, H. Song, J. Park, and K. M. Kim, Self-clocking fast and variation tolerant true random number generator based on a stochastic mott memristor, Nat. Commun. **12**, 2906 (2021).

[3]	W. Park et al., Frequency Switching Neuristor for Realizing Intrinsic Plasticity and Enabling Robust Neuromorphic Computing, Advanced Materials (2025).

[4]	W. A. Borders, A. Z. Pervaiz, S. Fukami, K. Y. Camsari, H. Ohno, and S. Datta, Integer factorization using stochastic magnetic tunnel junctions, Nature **573**, 390 (2019).

[5]	W. Whitehead, Z. Nelson, K. Y. Camsari, and L. Theogarajan, CMOS-compatible Ising and Potts annealing using single-photon avalanche diodes, Nat. Electron. **6**, 1009 (2023).

[6]	S. Cheong et al., Multi-State Probabilistic Computing Using Floating-Body MOSFETs Based on the Potts Model for Solving Complex Combinatorial Optimization Problems, (2026).

[7]	A. A. Mekawy, H. Moussa, G. Xu, M.-A. Miri, and A. Alù, Enabling a Potts machine with phase tristability, Phys. Rev. Appl. **24**, 034034 (2025).

[8]	C. Duffee, J. Athas, A. Grimaldi, D. Volpe, G. Finocchio, E. Wei, and P. Khalili Amiri, P-dits: Probabilistic d-dimensional bits for extended-variable probabilistic computing, Phys. Rev. Appl. **24**, 044077 (2025).
